# Male partners’ views of involvement in maternal healthcare services at Makhado Municipality clinics, Limpopo Province, South Africa

**DOI:** 10.4102/phcfm.v8i2.929

**Published:** 2016-05-06

**Authors:** Kenneth Nesane, Sonto M. Maputle, Hilda Shilubane

**Affiliations:** 1 Department of Advanced Nursing, University of Venda, Thohoyandou, South Africa

## Abstract

**Background:**

Male partners have a strong influence on pregnant partners’ health and their access to care. Their involvement is critical in the delivery and uptake of maternal healthcare services and improving maternal and child health outcomes.

**Aim:**

The study sought to determine male partners’ views on their involvement in maternal healthcare services.

**Setting:**

The Makhado Municipality’s Kutama, Madombidzha and Vleifontein clinics.

**Methods:**

A qualitative study design, which is exploratory, descriptive and contextual in nature, was used. The population comprised 15 men whose partners had been pregnant within the last 2 years. A non-probability, purposive sampling procedure was used. Data were collected via in-depth individual interviews using a voice recorder and an interview schedule guide. Tesch’s open coding method was used to analyse data.

**Results:**

The findings revealed one major theme, namely that maternal health issues are viewed as a woman’sdomain; and three sub-themes: culture and participation in childbirth, male partners’ employment status, and male partners’ unwillingness to participate in maternal health issues.

**Conclusions:**

The involvement of male partners in maternal healthcare services, and further research in promoting this activity, should be proposedto policymakers.

## Introduction

A maternal healthcare service comprises a wide range of health services provided to mothers before pregnancy, during pregnancy, during labour, and after giving birth. These health services include: preconception care, antenatal care (ANC), prevention of mother-to-child transmission (PMTCT) of HIV, safe delivery (intrapartum care), postnatal care (PNC), and emergency obstetric care/management of obstetric complications. However, for the purposes of the present study, maternal health services refers to ANC, delivery and PNC.

Most maternal and child health (MCH) programmes seek to address the health needs of women and children by engaging and educating pregnant women and mothers in appropriate care-seeking and care-giving practices for themselves and their children.^1^ The present study focuses on women, and their tendency to think about pregnancy, childbirth and child health as ‘women’s business’. This has often led to men being excluded from areas and services where they could learn more about reproductive, MCH matters.^1^ Kinanee^2^ concurs with this assertion, in indicating that maternal health issues have traditionally been predominantly seen and treated as a purely feminine matter. In the Vhembe District, Limpopo Province, pregnancy and childbirth has been a woman’s domain, and maternal healthcare services have focused on women, with very little male involvement.

Men tend to be decision-makers within families and often govern behaviour regarding the use of contraceptives, the availability of nutritious food, women’s workload, and the allocation of money, transport and time for women to attend health services. In addition, men’s behaviour influences the reproductive health of both men and women and the health of their children. Yet men are often unable to make informed choices because they have not been included in reproductive, MCH services and education.^3^ The failure to incorporate men in maternal health promotion, prevention and care programmes by policy makers, programme planners and implementers of maternal health services has had a serious impact on the health of women and the success of these programmes.^4^ Yet most African women are still unaware of their fundamental rights to health, and they continue to suffer from socioeconomic discrimination and unwanted pregnancies, which are harmful to their health.^5^ Women tend to have less education and fewer job opportunities than men have, which influences their maternal health-seeking behaviour and maternal health outcomes. Greater male involvement in maternal health programmes may help to reduce unintended, unwanted pregnancies and the transmission of sexually transmitted infections as well as improve child survival.^6,7^

### Objectives

The objectives of the study were to explore and describe the views of male partners on their involvement in maternal healthcare services provided at clinics of the Makhado Municipality, Limpopo Province.

## Research methods and design

### Study design

An exploratory descriptive design was used which helped the researcher to understand the phenomenon studied from the participants’ viewpoint in the context where the activities took place.^8^ A descriptive design facilitated data gathering pertaining to the views of male partners on their involvement in maternal healthcare services.^9^

### Study population and sampling strategy

The study population included all men who had had pregnant partners in the past 2 years who had attended the Kutama, Madombidzha and Vleifontein clinics of Makhado Municipality in Limpopo Province. The study setting was purposively selected. A purposive sampling technique was used to select five participants from each clinic on the basis that they possessed the characteristics of interest to the researcher.^9^

### Data collection

Data collection was done by the researcher at participants’ homes. The unstructured individual interviews were conducted to collect data from the 15 participants until saturation was reached. The following main question was asked: ’Can you kindly describe your views related to the involvement that needs to occur for male partners during maternal health care services.’ Other probing questions followed after the participants’ responses to the central question. Field notes were taken to capture aspects such as facial expressions that could not be recorded by the voice recorder.^9,10^

### Data analysis

Tesch’s open coding method was used to analyse data. Data analysis involved reading and re-reading verbatim transcriptions of all interviews sessions to acquire a sense of the whole. All topics were listed, and themes and sub-themes were classified and codes allocatedto them, and field notes were also coded.^11^

### Trustworthiness

To ensure trustworthiness in the study, the criteria of credibility and transferability^10^ were adhered to. Credibility was ensured by triangulation of data collection methods whereby a voice recorder was used to capture all interview sessions, and field notes were written to supplement what was not captured by the recorder. Prolonged engagement in the study field, where the researcher stayed for 3 months whilst accompanying male partners, also provided a credible data source. Transferability was ensured by thick descriptions of the research methodology, and data collection and purposive sampling which were used to include participants’ views.

## Ethical considerations

Permission to collect data was granted by the nurse managers in charge of the healthcare facilities where the research was conducted. Ethical approval was obtained from the University of Venda’s research ethics committee (SHS/14/PDC/08/1411). Informed consent forms were signed by all participants before commencement of interview sessions to confirm voluntary participation. The purpose of the study was outlined to all participants at the beginning of each interview session. Anonymity and confidentiality were ensured throughout the study.

## Results

Table 1 summarises biographical data of the participants.

**TABLE 1:** Biographical data of participants.

Male partner	Age (years)	Education	Ethnic group	Employment status	Marital status
1	33	Computer certificate	Venda	Employed	Married
2	27	Grade 12	Venda	Employed	Single
3	37	Grade 11	Venda	Unemployed	Married
4	30	Agriculture diploma	Venda	Unemployed	Married
5	34	Teacher’s diploma	Venda	Employed	Single
6	36	Grade 12	Venda	Unemployed	Single
7	26	Grade 12	Venda	Unemployed	Single
8	29	Grade 12	Venda	Unemployed	Single
9	28	Grade 11	Venda	Unemployed	Single
10	35	Computer certificate	Venda	Employed	Married
11	26	Grade 12	Venda	Unemployed	Single
12	27	Grade 10	Venda	Unemployed	Single
13	30	Grade 12	Venda	Employed	Married
14	32	Grade 9	Venda	Unemployed	Single
15	36	Grade 11	Venda	Unemployed	Married

Table 2 presents the theme and two sub-themes that emerged from the data.

**TABLE 2:** Main theme and sub-themes.

Theme	Sub-themes
Maternal health issues are viewed as women’s matters.	1.1. Culture and participation in childbirth. 1.2. Male partner employment status. 1.3. Unwillingness to participate in maternal health issues.

## Discussion

The findings below include a summary of the events and interviews, with discussion and quoted statements that are supported by the literature as views of male partners in involvement in maternal healthcare services.

### Theme: Maternal health issues viewed as women’s matters

The results revealed that lack of knowledge about maternal health issues led to non-participation and fear of the unknown by male partners. This finding is supported by Jooste^12^ who stated that, in general, men do not accompany their female partners when they attend these clinics, nor do they participate fully in the antenatal and PNC of their partners. In the present study, male partners would sit in their car, waiting for their women for more than an hour, complaining because they did not know the services which are rendered at the clinic and the expectations from them. This observation was supported by one participant when saying:

‘When my wife was pregnant, there is not much expected of me to do because those things are for women. I only help with transport, and I know that I must do some minor things at home which I think might be hard for her since she is pregnant.’

Women have to generally wait for a long time before receiving ANC services because of burdensome administrative procedures that result in poor patient/client care in health facilities. Men, who frequently are in a paid workforce, are usually not in a position to spend virtually the entire day participating in ANC services.^13^ Husbands’ interest levels and their attempts to support pregnancy health were relatively high, whilst low knowledge levels appeared to pose a significant obstacle to positive involvement. In general, all the participants’ knowledge surrounding pregnancy was limited, particularly in relation to complications or danger signs during pregnancy. Knowledge levels did not differ amongst male partners according to their presence or not at ANC.^13^ Sub-themes that emerged were: culture and participation in childbirth; male partners’ employment status; and unwillingness to participate in maternal health issues.

### Sub-theme 1.1: Cultural guidance in childbirth participation

Participants indicated that their culture, Tshivenda, does not allow them to participate in maternal healthcare services. This was expressed by one participant as follows:

‘My honourable one, what do I want inside because nurses learnt about their work. Our Tshivenda culture does not allow that, a male in the delivery room? [*exclamation*] Where a woman is giving birth … [*shaking the head*] … No no.’ (Participant 3)

Culture is perceived as a factor that affects male partners’ involvement in maternal healthcare services. All male partners who participated in the study were of the Tshivenda ethnic group. Contextual factors, such as paternal age, ethnicity, education, and family decision-making patterns, have been shown to influence male involvement in maternal health.^14,15,16,17^

Another participant said:

‘The elders are the ones who take care of the child, and they are very strict when it comes to the child. I am not allowed to carry my child before they do the rituals, and I am not allowed to get into the room where my wife and child are in.’ (Participant 2)

In many cases, it has been observed that men reject participation in female-oriented health services, encountering cultural as well as structural barriers such as a unit that accommodates more than one woman. Literature in this regard shows that service providers sometimes play a crucial role in barriers to men’s participation in ANC services.^18,19^

### Sub-theme 1.2: Male partners’ employment status a determination for non-involvement

Participants cited long distances from workplace to home as a factor that contributed to their non-participation in maternal healthcare services. For male partner involvement to take place, short distances are necessary and, if labour occurs spontaneously, partners may not be available. Most participants worked far from their homes, thus hindering their involvement in maternal healthcare services.

This finding was supported by the following quote:

‘About ANC, she goes there alone. I accompany her only when I am present but most of the time she goes there alone, and she will meet others on her way to the clinic. How do I involve myself with her antenatal care? It is because there is nothing I shall be doing there. Most of the times I was at work and the clinics are always over crowded by women and I don’t like it.’ (Participant 4)

Women wait for a long time before receiving ANC services because of burdensome administrative procedures which result in poor patient/client care in health facilities. Byamugisha^20^ indicated that men, who frequently are in the paid workforce, are often not in a position to spend virtually the entire day participating in ANC services. Traditionally, maternal health issues have predominantly been seen and treated as a purely feminine matter; this is because women fall pregnant and give birth. Although men’s participation in MCH services is low, they play a vital role in the safety of their female partners’ pregnancy and childbirth:^21^

‘We as men do not have knowledge of how important it is to enter with her in the consultation room or labour unit when she is about to give birth. The other thing is that we as men believe in different things; other people’s belief do not allow them to enter in the labour ward. That is why it is difficult to find men involving themselves. I work far from home.’ (Participant 3)

Dan et al.^22^ described the ideal father as one who was available, easily reached, accessible and considerate. Most men were willing to learn about their expected roles during childbirth and were eager to support their partners during this time. They found the health system unwelcoming, intimidating and unsupportive. Suggestions to improve men’s involvement included creating more awareness for fathers, male-targeted antenatal education and support, and changing provider attitudes.^22^ Kinanee^2^ mentioned that other barriers to the husband’s involvement included embarrassment in learning about pregnancy health, work obligations, hospital restrictions on the husband’s entrance into most areas of the hospital, and communication barriers between husbands and wives. However, there are different views with regard to what other European countries do. In Spain, paternity leave is 2 weeks, non-transferable, and 100% paid, which is used by more than 80% of parents.^23^ The rights of both parents have not yet been equalised in suitable conditions formaximising the chances of male responsibility, because paternal leave still differs widely from maternity leave, which is currently 16 weeks paid at full salary.^24^

### Sub-theme 1.3: Portrait excuses for unwillingness to participate in maternal health issues

In the present study, some participants raised the point that they were not willing to participate in maternal healthcare services because they viewed childbirth as ‘women issues’. To some male partners, maternal health was indeed seen as a women’s issue and that they did not have to take part. This view was supported by the following statement:

‘No! Nurses do not refuse a person to get in, but I feel it myself that that place is not a place to play, it is for women, there is nothing I can do there [*laugh*].’ (Participant 1)

An important exception in Africa was a study conducted in Nigeria, where limited birth preparedness and participation by men in a patriarchal society was reported, and a study in Uganda in which spousal influence was identified to be amongst the main factors affecting the choice of delivery place.^15^ Gabrysh^25^ found that women’s use of maternal health services was associated with higher education of the husband, probably owing to educated men’s positive attitudes toward modern healthcare, wider knowledge of the benefits of skilled attendance at birth, and perhaps less restriction on wives’ movements. This finding was supported by the following excerpt:

‘Normally when I get to the hospital I participate but reluctant to get inside the consultation or maternity unit because there were other women who were in labour and I can’t get inside because some would be naked.’ (Participant 6)

Chattopadhyay^26^ indicated that men were not always encouraged to be involved during pregnancy and childbirth in the South-Asian context. For example, men in Nepal are typically discouraged from involvement with pregnancy and childbirth. Literature in this regard shows that service providers sometimes play a crucial role in creating barriers for men to participate in ANC services.^18^ However, in the South African context; male partners are encouraged to be involved in maternal health issues.^27^

## Recommendations

The following strategies were recommended to facilitate promoting the involvement of male partners and to address factors that contribute to non-participation of male partners in maternal health care services in Makhado Municipality.

Primary healthcare nurses, in their role of facilitating male partner involvement, need to motivate the male partner by ensuring that he realises the importance of active involvement in maternal health services. For example, attending maternal health facilities with their partners, and assisting partners to understand their problems and needs in totality, will lead to greater understanding of their families and the community in general. The clinics at Makhado Municipality should identify innovative ways of implementing the policy of male involvement in pregnancy and childbirth in order to effectively engage men who are keen to be involved in the healthcare of their partners. These might involve health education of men who escort their partners to antenatal clinics, and on expected roles during pregnancy and childbirth. The related clinic should train healthcare providers in customer care, and have waiting rooms where men are welcome, provided with information on their spouses, given education on health needs and specific roles in pregnancy and childbirth, and highlighting the importance of these rolesin positive pregnancy outcomes. Further needs are to assist the facilities to establish community outreach, clinic-based education and couple-oriented counselling interventions. Such steps would improve male involvement, as would the distribution of information, education and communication materials on relevant maternal health issues. Informal peer information-sharingwould also encourage the male initiative. Men could be invited to participate in maternal healthcare and to then inform their peers about their experiences and encourage them to participate.

Cultural factors were identified as barriers to male involvement. Studies have reported negative perceptions toward men attending ANC services. The influence of local cultural lore showed that effective health interventions should take into account traditional beliefs and customs in order to achieve health goals. Midwives should provide culturally congruent care, and they should be able to care for, and communicate with, patients who belong to different cultures during maternal healthcare. Community health workers (CHWs) should be encouraged to conduct community outreachin villages to disseminate messages about male involvement, and to collaborate with community leaders on how to approach the men.

## Conclusion

Inadequate knowledge, cultural factors and lack of appropriate services were found to have negatively influenced male participation and involvement in maternal healthcare services.^28^ Findings of the present study are that men still view maternal health as women’s issues. Although men are not direct beneficiaries of safe motherhood services, their understanding, participation, involvement and support is crucial in order for women to access basic reproductive health services.
